# Characterization of complete mitochondrial genome of *Jaydia lineata* (Perciformes, Apogonidae)

**DOI:** 10.1080/23802359.2018.1511844

**Published:** 2018-10-29

**Authors:** Bingjian Liu, Yifan Liu, Kehua Zhu, Liqin Liu, Lihua Jiang, Li Gong, Zhu Liu, Weinan Zhang, Wen Duan, Zhenming Lü

**Affiliations:** a National Engineering Laboratory of Marine Germplasm Resources Exploration and Utilization, Zhejiang Ocean University, Zhoushan, PR China;; b Laboratory for Marine Fisheries Science and Food Production Processes, Qingdao National Laboratory for Marine Science and Technology, Qingdao, PR China;; c National Engineering Research Center for Facilitated Marine Aquaculture, Marine science and technology college, Zhejiang Ocean University, Zhoushan, PR China

**Keywords:** *Jaydia lineata*, mitogenome, phylogenetic tree

## Abstract

The complete mitogenome of *Jaydia lineata* was sequenced, which is a closed double-stranded circular molecule of 16,506 bp, and we analyzed the main features in terms of the genome organization, gene arrangement, and codon usage. The overall base composition includes C(28.5%), A(27.2%), T(26.7%), and G(17.6%). Moreover, the 13 protein-coding genes encode 3797 amino acids in total, 12 of which use the initiation codon ATG except COI uses GTG. Most of them have TAA as the stop codon, whereas ND3 ends with TAG, and three protein-coding genes (COII, ND4, and Cytb) use ended with the incomplete stop codon represented as a single T. The phylogenetic tree based on the neighbour-joining method was conducted, using a concatenated set of 12 protein-coding genes, and adding nine other species of Perciformes, which could be a useful basis for management of this species.

*Jaydia lineata* inhabits sandy from coastal inlets to a depth of 100 m from Uchiura Bay in Hokkaido, Japan, to the South China Sea and Indo-West Pacific. Because of the significant changes in the structure of the fishery in the East China Sea, the status and role of small fish in the energy transformation of the marine food chain is becoming more and more important. In this article, we described the characterization of complete mitochondrial genome of *J. lineata* and explored the phylogenetic relationship within Perciformes, which were expected to facilitate future studies on taxonomic resolution, population genetic structure, and phylogenetic relationships.

Specimens of *J. lineata* were sampled by commercial bottom trawling in ZhouShan, China (29°59′33″N; 122°12′28″E) and stored in laboratory of Zhejiang Ocean University with accession number 20150826XT22.

The mitogenome of *J. lineata* is a closed double-stranded circular molecule of 16,506 nucleotides (GenBank Accession Number: MH244441), consisting of 13 PCGs, 22 tRNA genes, two rRNA genes, one replication origin and a control region, this feature was similar to the typical mitogenome of other vertebrates (Zhu, Gong, Jiang, et al. 2018; Zhu, Gong, Lü, et al. 2018; Zhu, Lü, Liu, et al. 2018). The overall base composition is 27.2, 26.7, 28.5, and 17.6% for A, T, C, and G, respectively, with a slight AT bias 53.9%. The 13 PCGs genes encode 3797 amino acids in total, similar to the typical mitogenome of vertebrates, all the PCGs use the initiation codon ATG except COI use GTG (Miya et al. 2001). Most of them have TAA or TAG as the stop codon, except COII, ND4, and Cytb use an incomplete stop codon T, these incomplete termination codons were presumably completed as TAA *via* post-transcriptional polyadenylation (Ojala et al. 1981). The 12S rRNA and 16S rRNA are 955 and 1679 bp, respectively. The tRNA genes were generated using the programme tRNAs-can-SE (Lowe and Eddy 1997), all of them can fold into a typical cloverleaf structure except tRNASer (AGY), which lacks a dihydrouridine arm. As in most vertebrates, two non-coding regions are found in the *J. lineata* mitogenome (Jiang et al. 2018), the OL is located in a cluster of five tRNA genes (WANCY), which has the potential to fold into a stable stem-loop secondary structure, with a stem formed by 13 paired nucleotides and a loop of 13 nucleotides; the CR, with 847bp, is located between tRNA-Pro and tRNA-Phe, and the core sequence of the terminal-associated sequence (ACATATATGT) was identified in the CR of *J. lineata* which is identical to that in other teleostean mitogenomes (Zhang et al. 2013).

To explore the phylogenetic position of this *J. lineata*, a phylogenetic tree was constructed based on the NJ analysis of twelve PCGs encoded by the heavy strand. The results of this study support *J. lineata* has a closest relationship with *Apogon semilineatus*, highly supported by a bootstrap value of 100 (Figure 1), which is in accord with the previously reported study (Nishida et al. 2006).

**Figure 1.**
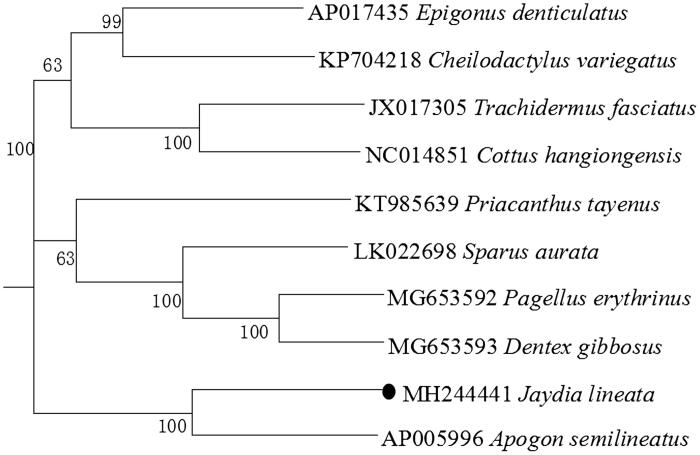
Neighbour-joining (NJ) tree of 10 Perciformes species based on 12 PCGs. The bootstrap values are based on 1000 resamplings. The number at each node is the bootstrap probability. The number before the species name is the GenBank accession number. The genome sequence in this study is labelled with a black spot.
